# Advances in the anatomic study of the interscapular region of the cat

**DOI:** 10.1186/s12917-015-0562-y

**Published:** 2015-10-05

**Authors:** Maurizio Longo, Silvia Clotilde Modina, Andrea Bellotti, Mauro Di Giancamillo

**Affiliations:** Department of Veterinary Sciences and Public Health, Università degli Studi di Milano Italy, Via Celoria 10, 20133 Milano, Italy; Department of Health, Animal Science and Food Safety, Università degli Studi di Milano, Via Celoria 10, 20133 Milano, Italy

**Keywords:** Cat, Cadaver, Interscapular region, Cross sectional anatomy, Gross anatomy, Computed tomography, Magnetic resonance imaging, Double forelimb positioning, Feline injection-site sarcoma, Fibrosarcoma

## Abstract

**Background:**

New clinical oriented approaches are supported by the integration of advanced imaging techniques, e.g. computed tomography and magnetic resonance, with gross anatomy imaging. The interscapular region of the cat is a typical site of a highly invasive infiltrative pathology, i.e. Feline Injection-Site Sarcoma. Even if chemotherapy and radiotherapy have been considered as pre-surgical therapies, extensive surgery is still the recommended treatment. Evidence suggested that the relationships between muscles, infiltrative mass and adjacent musculoskeletal structures could change according to the forelimb positions: a fact to be duly considered while planning the surgical approach.

Anatomic and tomographic atlases provide only images of the interscapular region from cats positioned with their forelimbs extended cranially, which means that, they do not record musculoskeletal modifications due to the forelimb movements.

Aim of this study was to provide detailed images of the changes occurring in the musculoskeletal structures of the interscapular region of cats according to their forelimb position by comparing cross-sectional gross anatomy, computed tomography and magnetic resonance imaging.

**Results:**

We provide an atlas of normal cross-sectional anatomy, computed tomography and magnetic resonance imaging of the interscapular region of the cat, from the fifth cervical vertebra to the fifth thoracic vertebra. We compare and couple the slices obtained both in flexed and extended forelimb positioning with the animal maintained in sternal recumbency.

**Conclusion:**

This study shows a new and dynamic way to investigate the interscapular region of the cat and provides anatomical references for in vivo computed tomography and magnetic resonance imaging, considering changes in the muscular form according to the forelimb positioning. We believe that an in-depth anatomical knowledge of the interscapular region may be helpful to approach the study of any pathology located there and, in particular, to set up an appropriate therapy for the feline injection-site sarcoma.

## Background

The interscapular region is characterized by several layers of muscles held together by a complex myofascial system, often infiltrated and covered by adipose tissue, which allows their mutual movement, contributing to stabilize the scapula against external forces and preventing its displacement or rotation [1, 2].

In cats, the region, though extensively described in dogs [1], has been considered mainly with a comparative approach [2–5]. Traditional anatomical atlases and textbooks typically describe the musculature of the interscapular region in standing animals with forelimbs in neutral position, considering the muscles in their superficial and deep layers [1–7]. Conversely, anatomic tomographic images from scientific articles generally refer to animals in sternal [6–8] or dorsal recumbency [9, 10], even though sternal recumbency with forelimbs pulled cranially is indicated as the standard protocol [11]. Moreover, common literature considers only a static approach describing gross and/or sectional anatomy and/or tomographic anatomy only in a fixed position; not considering the high level of mobility of the interscapular region can induce significant change in the appearance of muscles during dissection procedures and/or image acquisition, depending on the position assumed by the forelimbs.

The only two studies describing specifically the dorsal muscles in cat, including the interscapular region, were published by Sami et al. [9, 10]. They compare cross sectional gross anatomy and computed tomography (CT) [9], cross sectional gross anatomy and magnetic resonance imaging (MRI) [10]. Currently, these works represent the only guidelines for the in toto study of the anatomy on the feline thorax and abdomen.

Cats commonly receive subcutaneous injections in their interscapular region, which has been widely described as a condition that may contribute to the growth of mesenchymal tumors referred to the group of Feline Injection-Site Sarcomas (FISS) [12–19].

FISS are characterized by an aggressive behaviour towards perilesional tissues, a low distant metastatic potential and a high grade of local recurrence [12–14, 17].

Though chemotherapy and radiotherapy have been considered as pre-surgical therapies also for this type of tumor [13, 14], radical and extensive surgery is still the treatment of choice [12–14, 17].

Being the surgical excision highly invasive and seriously compromising the patient’s health [12], CT and MRI have been recently introduced into clinical planning for a better definition of the relationship between neoplastic lesions and surrounding soft and hard tissues. As a consequence of the high mobility of the interscapular region, it has been postulated that the mass could change its relationship with surrounding tissues depending on the forelimb position during CT scan [12].

A fact to duly consider while planning the indicated surgical approach: in fact, an improper description of the neoplastic lesion could compromise both treatment and follow-up [12, 14] for the patients.

The aim of this work is to provide an atlas of normal cross-sectional gross anatomy, CT and MRI anatomy of the interscapular region of the cat with the forelimbs extended cranially and caudally along the body, in sternal recumbency. In order to obtain a more accurate description of the region, all the images were correlated by comparing the different features assumed by soft and hard tissues, in the two different positioning.

## Methods

Three adult domestic shorthaired cats (subjects A, B, C) supplied by owners prior written informed consent and died for causes unrelated to the present study (i.e. on natural or euthanized according to good clinical practice guidelines) were enrolled in our Veterinary Teaching Hospital for educational purpose. No specimen showed any musculoskeletal lesion in the interscapular region.

### CT and MRI images acquisition

Cadavers were stored at 4 °C for 24 h, then a complete CT and MRI scan were performed in subject A (5 kg body weight) and in subject B (5 kg body weight), respectively. CT images were obtained employing a single slice CT unit model Picker PQ2000S (Philips Healthcare, Monza, Italy) in helical mode by 3 mm thickness contiguous slices, with a pitch of 1, 120 kV, 150 mA, 1 s of tube rotation and an average scan time of 35 s. Images were assessed using certified software Osirix^Pro^ 64-bit (Aycan Medical Systems, LLC, Rochester, NY). MRI scans were performed using a low field MRI unit (0,2 T) model Vet-MR (Esaote S.p.A, Genova, Italy). On MRI, sections of 3 mm thickness were acquired with an average total acquisition time of 50 min. In all specimens T1-weighted, T2-weighted and High Resolution Gradient Echo sequences were employed.

Both CT and MRI scans were performed with the following protocols: 1) sternal recumbency and volumetric acquisition with the forelimbs pulled cranially; 2) sternal recumbency and volumetric acquisition with the forelimbs flexed caudally along the body, obtained immediately after the first acquisition.

Subject C (4 kg body weight) was submitted to a CT scan and, immediately after, to an MRI scan, with the following protocol: CT images were obtained by 1.25 mm thickness contiguous slices employing a 16-slice CT unit model Brightspeed-16 (General Electric Healthcare, Milano, Italy). MRI images were obtained by 4 mm thickness employing the above-mentioned low-filed MRI unit.

For both CT and MRI, the field of view was extended from the fifth cervical vertebra (C5) to the fifth thoracic vertebra (T5). An unmovable marker placed on the body of the animal identified the top and the bottom of the study. All the images were evaluated in all three axes. On CT study, dorsal and sagittal planes were obtained by multiplanar reconstructions, while on MRI study they were directly acquired.

### Anatomic images acquisition

In order to collect gross anatomical sections at the same level of the tomographic slices, at the very end of every tomographic study, subjects A and B were placed in a plastic case of polystirene (dimensions of the case: 55 × 36 × 19 cm) and kept in a−18 °C freezer until solid, strictly maintained in the same position assumed in CT or MRI study by wooden sticks and synthetic resin sponges.

The frozen cadavers obtained in a rectangular shape in the plastic case were sectioned along the transverse plane with an electric band saw. Six mm-thick sections, extended from C5 to T5, previously identified by the markers, were obtained. Consequently, each section included two adjacent CT or MRI slices, in order to obtain the best correspondence between cross-sectional cuts and imaging slices.

Frozen slices were numbered and gently cleaned of debris with cold water and light brushing. The two surfaces of each section were photographed instantaneously (i.e., before thawing) with a digital Nikon D90 photographic camera, with a 300 dpi resolution.

### Organisation of the tables

Photographs were examined and identifiable anatomic structures were labelled with the available anatomical references [1–5, 9, 10]. The terminology adopted was chosen in accordance to the *Nomina Anatomica Veterinaria* (2012). For each anatomical slice, a corresponding CT and MRI slice was chosen on the basis of their comparable appearance and presented in flexed and extended positioning, considering as reference pivot the vertebral bodies.

Identified structures were subsequently located on the anatomical sections and on CT and MRI slices. Afterward, the list of the identified structures was evaluated against CT and MRI images from cadaver C.

## Results

### Atlas of matched cranio-caudal sections of the interscapular region of a normal cat obtained by CT, MRI and cross-sectional anatomy, in double positioning of the forelimbs

**Fig. 1:**
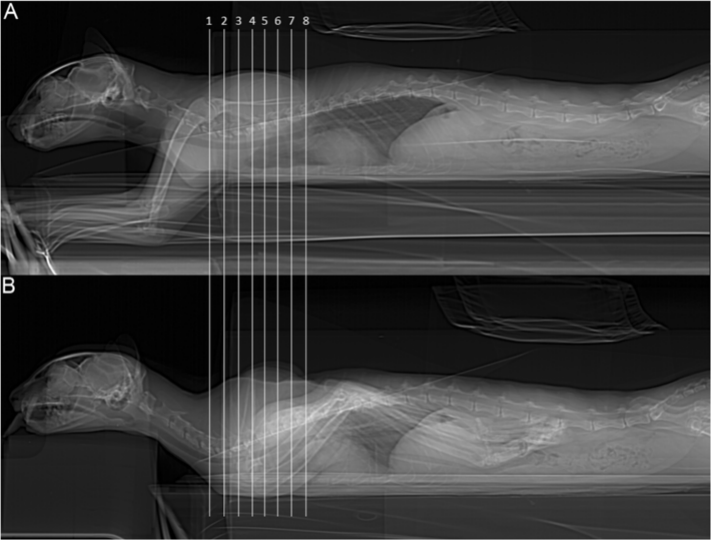
Total-body scanogram with extended (**a**) and flexed (**b**) forelimbs obtained with a 16 slice CT. Numbered lines indicate the approximate levels of each anatomic slice of the frozen cadaver and the two corresponding contiguous CT and MRI sections

**Fig. 2:**
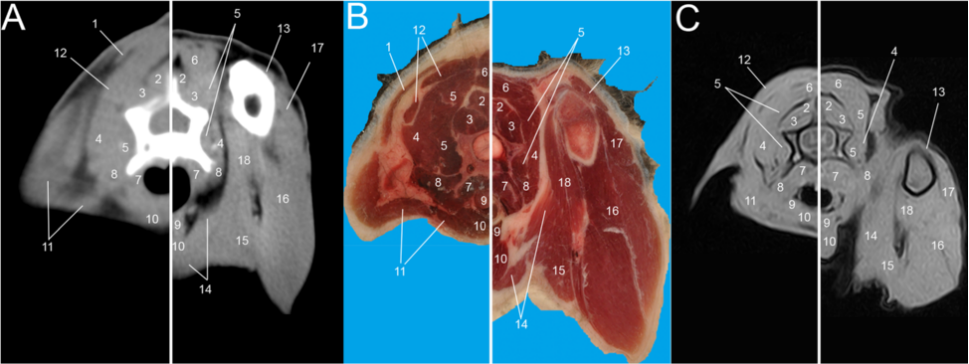
Single slice CT with 400 WW and 40 WL (**a**), Anatomical (**b**) and low field T1-weighted MRI (**c**) slices obtained at the level of C5. Images show flexed forelimb positioning on the left and extended forelimbs positioning on the right: 1. *M. trapezius*, 2. *M. spinalis*, 3. *M. multifidus*, 4. *M. serratus ventralis*, 5. *M. longissimus cervicis et capitis*, 6. *M. splenius*, 7. *M. longus colli*, 8. *M. scalenus*, 9. *M. sternohyoideus*, 10. *M. sternocephalicus*, 11. *M. brachiocephalicus*, 12. *M. rhomboideus*, 13. *M. brachialis*, 14. *M. pectorales superficialis*, 15. *M. pectorales profundus*, 16–17. *M. triceps brachii*, 18 *M. biceps brachii*

**Fig. 3:**
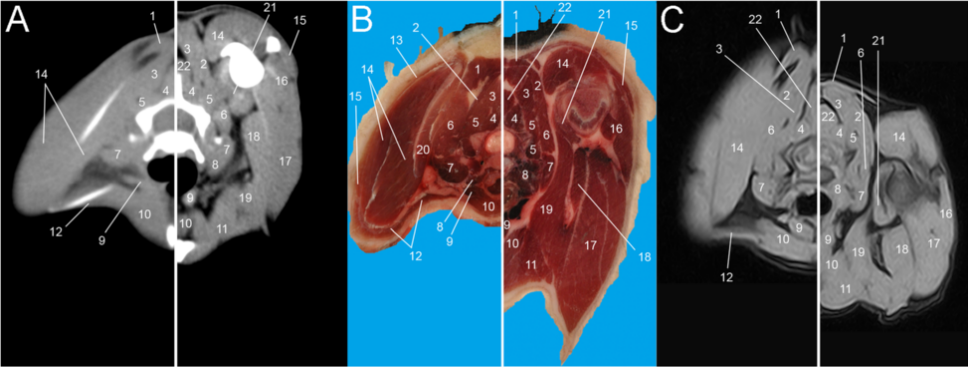
Single slice CT with 400 WW and 40 WL (**a**), Anatomical (**b**) and low field T1-weighted MRI (**c**) slices obtained at the level of C6. Images show flexed forelimb positioning on the left and extended forelimbs positioning on the right: 1. *M. rhomboideus*, 2. *M. splenius*, 3. *M. longissimus capitis*, 4. *M. multifidus*, 5. *M. longissimus cervicis*, 6. *M. serratus ventralis*, 7. *M. scalenus*, 8. *M. longus capitis*, 9. *M. sternohyoideus*, 10. *M. sternocephalicus*, 11. *M. pectorales superficialis*, 12. *M. brachiocephalicus*, 13. *M. trapezius*, 14. *M. supraspinatus*, 15. *M. omotransversarius*, 16. *M. deltoideus*, 17. *M. triceps brachii*, 18. *M. teres major*, 19. *M. pectorales profundus*, 20. *M. subscapularis*, 21. *M. biceps brachii*, 22. *M. spinalis*

**Fig. 4:**
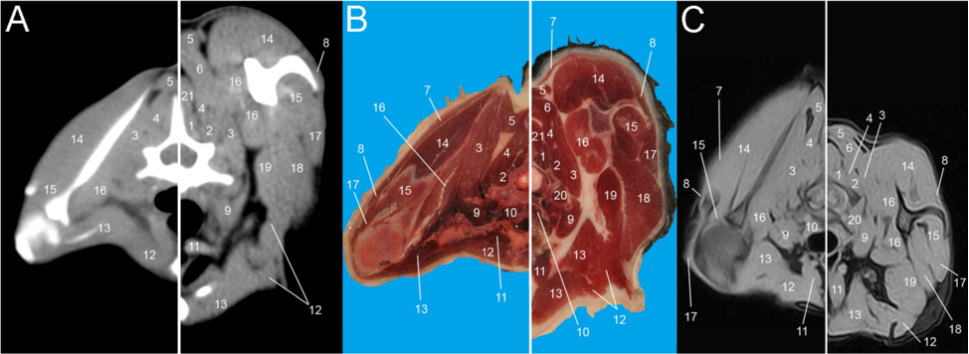
Single slice CT with 400 WW and 40 WL (**a**), Anatomical (**b**) and low field T1-weighted MRI (**c**) slices obtained at the level of C7. Images show flexed forelimb positioning on the left and extended forelimbs positioning on the right: 1. *M. multifidus*, 2. *M. longissimus cervicis*, 3. *M. serratus ventralis*, 4. *M. semispinalis*, 5. *M. rhomboideus*, 6. *M. splenius*, 7. *M. trapezius*, 8. *M. omotransversarius*, 9. *M. scalenus*, 10. *M. longus colli*, 11. *M. sternocephalicus*, 12. *M. pectorales superficialis*, 13. *M. pectorales profundus*, 14. *M. supraspinatus*, 15. *M. infraspinatus*, 16. *M. subscapularis*, 17. *M. deltoideus*, 18. *M. triceps brachii*, 19. *M. teres major*, 20. *M. longissimus capitis*, 21. *M. spinalis cervicis*

**Fig. 5:**
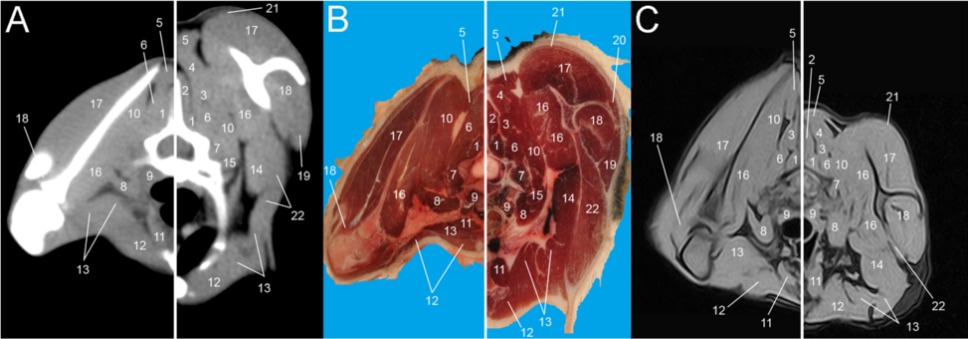
Single slice CT with 400 WW and 40 WL (**a**), Anatomical (**b**) and low field T1-weighted MRI (**c**) slices obtained at the level of T1. Images show flexed forelimb positioning on the left and extended forelimbs positioning on the right: 1. *M. multifidus*, 2. *M. spinalis cervicis*, 3. *M. longissimus capitis*, 4. *M. splenius*, 5. *M. rhomboideus*, 6. *M. longissimus cervicis*, 7. *M. complexus*, 8. *M. scalenus*, 9. *M. longus colli*, 10. *M. serratus ventralis*, 11. *M. sternocephalicus*, 12. *M. pectorales superficialis*, 13. *M. pectorales profundus*, 14. *M. teres major*, 15. *M. iliocostalis*, 16. *M. subscapularis*, 17. *M. supraspinatus*, 18. *M. infraspinatus*, 19. *M. deltoideus*, 20. *M. omotransversarius*, 21. *M. trapezius*, 22. *M. triceps brachii*

**Fig. 6:**
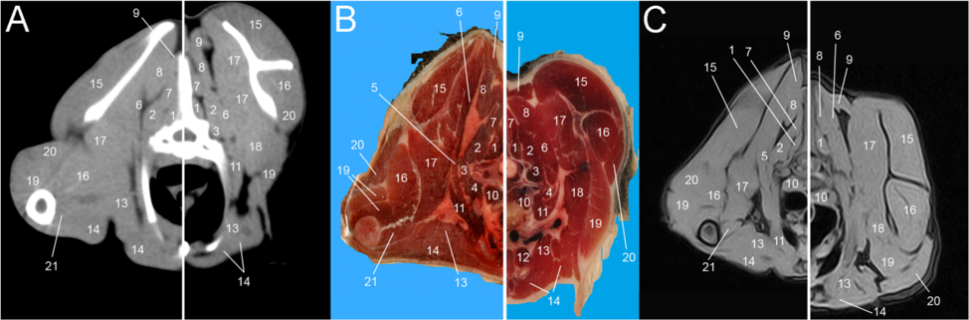
Single slice CT with 400 WW and 40 WL (**a**), Anatomical (**b**) and low field T1-weighted MRI (**c**) slices obtained at the level of T2. Images show flexed forelimb positioning on the left and extended forelimbs positioning on the right: 1. *M. multifidus*, 2. *M. longissimus cervicis*, 3. *M. longissimus thoracis*, 4. *M. iliocostalis*, 5. *M. serratus dorsalis*, 6. *M. serratus ventralis*, 7. *M. spinalis thoracis*, 8. *M. rhomboideus*, 9. *M. trapezius*, 10. *M. longus colli*, 11. *M. scalenus*, 12. *M. sternothyroideus*, 13. *M. pectorales profundus*, 14. *M. pectorales superficialis*, 15. *M. supraspinatus*, 16. *M. infraspinatus*, 17. *M. subscapularis*, 18. *M. teres major*, 19. *M. triceps brachii*, 20. *M. deltoideus*, 21. *M. biceps brachii*

**Fig. 7:**
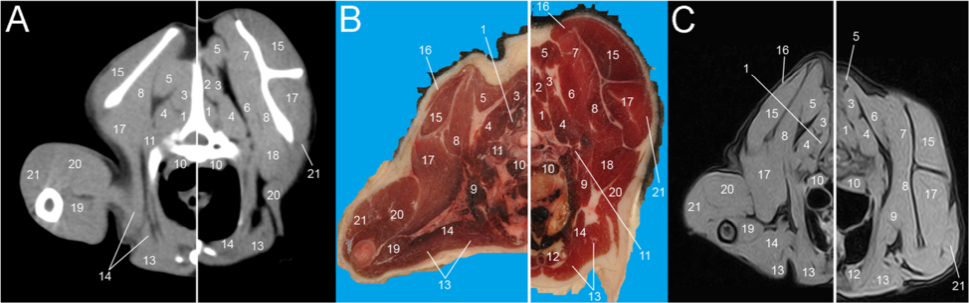
Single slice CT with 400 WW and 40 WL (**a**), Anatomical (**b**) and low field T1-weighted MRI (**c**) slices obtained at the level of T3. Images show flexed forelimb positioning on the left and extended forelimbs positioning on the right: 1. *M. multifidus*, 2. *M. spinalis thoracis*, 3. *M. longissimus cervicis*, 4. *M. longissimus thoracis*, 5. *M. rhomboideus*, 6. *M. serratus dorsalis*, 7. *M. serratus ventralis*, 8. *M. subscapularis*, 9. *M. scalenus*, 10. *M. longus colli*, 11. *M. iliocostalis*, 12. *M. sternothyroideus*, 13. *M. pectorales superficialis*, 14. *M. pectorales profundus*, 15. *M. supraspinatus*, 16. *M. trapezius*, 17. *M. infraspinatus*, 18. *M. teres major*, 19. *M. biceps brachii*, 20. *M. triceps brachii*, 21. *M. deltoideus*

**Fig. 8:**
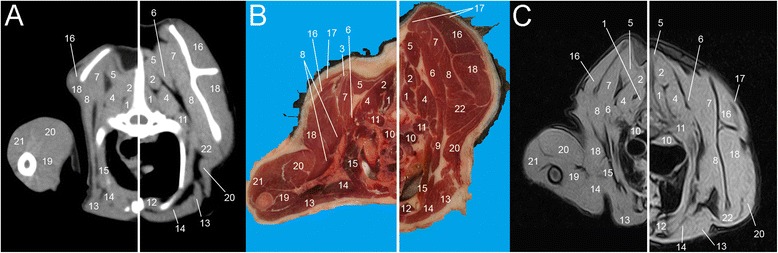
Single slice CT with 400 WW and 40 WL (**a**), Anatomical (**b**) and low field T1-weighted MRI (**c**) slices obtained at the level of T4. Images show flexed forelimb positioning on the left and extended forelimbs positioning on the right: 1. *M. multifidus*, 2. *M. spinalis thoracis*, 3. *Scapula*, 4. *M. longissimus thoracis*, 5. *M. rhomboideus*, 6. *M. serratus dorsalis*, 7. *M. serratus ventralis*, 8. *M. subscapularis*, 9. *M. scalenus*, 10. *M. longus colli*, 11. *M. Iliocostalis*, 12. *M. sternothyroideus*, 13. *M. pectorales superficialis*, 14. *M. pectorales profundus*, 15. *M. intercostalis*, 16. *M. supraspinatus*, 17. *M. trapezius*, 18. *M. infraspinatus*, 19. *M. biceps brachii*, 20. *M. triceps brachii*, 21. *M. deltoideus*, 22. *M. teres major*

**Fig. 9:**
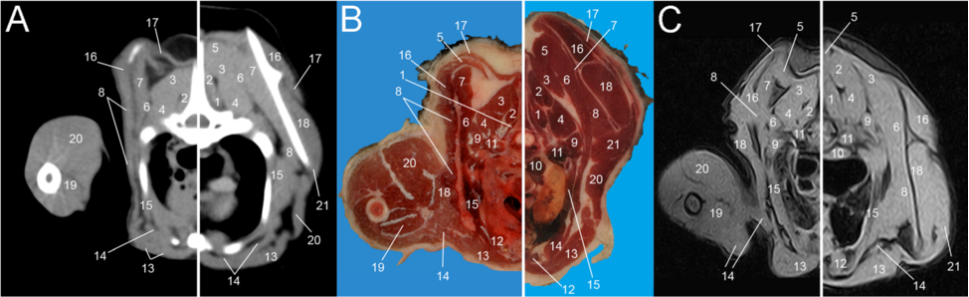
Single slice CT with 400 WW and 40 WL (**a**), Anatomical (**b**) and low field T1-weighted MRI (**c**) slices obtained at the level of T5. Images show flexed forelimb positioning on the left and extended forelimbs positioning on the right: 1. *M. multifidus*, 2. *M. spinalis thoracis*, 3. *M. longissimus cervicis*, 4. *M. longissimus thoracis*, 5. *M. rhomboideus*, 6. *M. serratus dorsalis*, 7. *M. serratus ventralis*, 8. *M. subscapularis*, 9. *M. scalenus*, 10. *M. longus colli*, 11. *M. iliocostalis*, 12. *M. sternothyroideus*, 13. *M. pectorales superficialis*, 14. *M. pectorales profundus*, 15. *M. intercostalis*, 16 *M. supraspinatus*, 17. *M. trapezius*, 18. *M. infraspinatus*, 19. *M. biceps brachii*, 20. *M. triceps brachii*, 21. *M. teres major*

Eight representative transverse combinations of CT, cross sectional anatomy and MRI slices with the forelimbs in cranial position and caudal position were obtained and then coupled. Figure 1a and b show lateral scanograms of the animal acquired immediately before the beginning of the scan in extended and flexed position respectively. The lines represent the levels of each CT, MRI and anatomic slice of the cadaver. Figures 2, 3, 4, 5, 6, 7, 8 and 9 show the cranio-caudal CT-slice (A), cross sectional anatomy (B) and MRI-slice (C) coupled in the flexed (on the left) and the extended (on the right) forelimbs position from C5 to T5.

All the muscles of the interscapular region were identified. Most of them were visible in both positioning; on the contrary, some muscles appeared only in flexed or extended positioning. As expected, the muscles *supraspinatus, infraspinatus, subscapularis, rhomboideus, serratus ventralis, semispinalis cervici* modified their aspect and their relationship to each other and to the scapulae, depending on the position assumed by the forelimbs. Substantial differences showed mainly remarkable between T1 and T5 as a direct consequence of the highest mobility of this tract (Figs. 5, 6, 7, 8 and 9).

When the forelimbs were maintained extended, in fact, the scapulae were aligned to the body, in a horizontal position (Fig. 1a). Consequently, the muscles *supraspinatus* and *infraspinatus.* followed the position of the scapulae, lying horizontally at the same distance. When the forelimbs were maintained flexed, the scapulae acquired a vertical position (Fig. 1b). In this case four events occur: 1) the muscles *supraspinatus, infraspinatus* and *subscapularis* followed the scapulae and assumed a vertical position in all images; therefore, scans were obtained on the major axis of every muscle; 2) a minor scapulae surface in the interscapular region was included; 3) the distance that divides the scapulae was not the same: bones showed closer to each other cranially than caudally; 4) a slight raising of the dorsal aspect of the scapulae resulted in a consequent raising of the muscles *romboideus*, *semispinalis capitus* and *serratus ventralis;* they appeared more defined, thinner and horizontally oriented if compared to the images obtained with extended forelimbs. In addition, with flexed forelimbs the adipose tissue and the myofascial organization were generally better visualised, appearing more opened and less compressed. These features were especially more identifiable on MRI and anatomical cross-sections.

## Discussion

The interscapular region is complex to investigate, with layers of overlaid muscles whose spatial relationships are difficult to evaluate from conventional imaging and anatomic references. Moreover, this region is characterized by high mobility that in some instances can over-or underestimate the relationships among muscles and between muscles and scapulae. A relevant aspect not considered in traditional literature together with a rapid identification of muscles themselves and of their spatial relationship with surrounding structures.

To our knowledge, this is the first atlas that provides an exhaustive description of the interscapular region of the cat and considers its dynamic behaviour. Data were obtained by comparing cross-sectional imaging of gross, CT and MRI anatomy, which permitted to better identify the musculoskeletal and myofascial components of the region while taking full advantage of the potential of the three methods.

From a technical point of view, this multiple approach represents a novelty in its considering both techniques simultaneously while previous studies in rabbits [20–23] dogs [6–8] and cats [9, 10] compared either cross-sectional anatomy and CT or cross-sectional anatomy and MRI. Further, unlike other studies [6–8, 10, 20–23], in our work the sections were 3 mm-thick on CT, 3 mm-thick on MRI, and approximately 6 mm-thick on cross-sectional anatomy, allowing improved comparison between gross anatomy slices and advanced imaging techniques. In this contest, the choice to maintain vertebral bodies as the reference pivot proved to be a useful tool in detecting anddescribing the anatomical structures.

All images obtained in this study were of excellent quality and no abnormalities due to sample preservation were observed.

As expected, soft tissue details were superior on MRI than on CT, and the sequence identified as High Resolution Gradient Echo showed better rendering of the morphology of the musculoskeletal structures. Low field MRI required a long acquisition time when 3 mm thickness was applied (total average acquisition time: 50 min); an aspect that might represent a problem only in clinical activities requiring a prolonged anaesthesia time, but not in our investigation carried on dead animals. Conversely, CT acquisition required a short resolution time; depending on the tomography used, it ranged from 35 to 15 s for single or multi-detector, respectively. In subject C we decreased thickness of the CT-slices down to 1.25 mm and no significant improvement in image quality was observed in a transverse plane. Opposite to CT, in the same subject we increased thickness of MRI slices up to 4 mm. Data demonstrated that the quality of images kept unchanged, but time taken was significantly shorter (total average acquisition time: 35 min), which makes clinical application more feasible.

The corpses of the cats were positioned in sternal recumbency with their forelimbs extended cranially or caudally along their bodies. As a consequence, the scapula and its related muscles moved against the chest wall in two different ways even thanks to the characteristic loose connective tissue presents between the limb and the trunk, where the axilla is located.

We observed that changes in the position of the scapula can modify the shape of the muscles, as clearly appreciable by CT and MRI and confirmed by gross anatomy images. Although Travetti et al. [12] provided exhaustive images of the interscapular region, it is important to underline that those images derived from cats affected by Feline Injection-Site Sarcoma (FISS), with manifest tumours in between their muscles. A situation where not only the movements of the limbs, but also the volume of the neoplastic masses may change the shape of the muscles implied, thus complicating the interpretation of the images. One of the key strengths of our work is that its images were derived from cats with no lesion, and therefore their consultation should avoid any misinterpretation.

In veterinary medicine_,_ sternal recumbency is the standard positioning commonly recognized for CT studies of the thoracic region [11]; however, in the cat, tomographic references available in literature are all obtained with animals positioned in dorsal recumbency [9, 10].

In animals, sternal recumbency should be preferred to dorsal recumbency to reduce possible lung congestion occurring in prolonged dorsal recumbency, with the forelimbs extended cranially to avoid including them in the visual field. In our study, sternal positioning associated to forelimbs either flexed along the body or pulled cranially was aimed at better visualizing the musculoskeletal structures by enhancing their mobility. Moreover, since double positioning made the myofascial system and tissue adipose infiltrations visible, the images acquired can support a more accurate estimation of the relationship between both infiltrating and neoplastic lesions and surrounding tissues.

In particular, in cats affected by FISS, a deep knowledge of the sectional anatomy of the region is mandatory in order to reduce post-surgical complications. It has recently been demonstrated that surgery time is the best predictor of wound healing complications and that they are influenced by excision pattern and mass size [24].

Currently, establishing the best relationship possible between a mass and its surrounding tissues while considering the mobility of the region is critical as extensive surgery, including ostectomy if necessary is still the treatment of choice [12–14, 17, 24].

Cost implication and availability of CT and MRI scanners are the most important limitations in the integrating cross-sectional anatomy. Moreover these techniques require a training and assessment in the interpretation of post-mortem imaging. In fact, by understanding the benefit and limit of each imaging technique we can employ CT and MRI to their maximal advantages.

## Conclusion

This work indicates a new approach to the interpretation of the interscapular anatomy of the cat in that it considers the dynamic behaviour of the region, a highly necessary component in the study of all pathologies, neoplastic and non-neoplastic, affecting it. Our claim was to provide radiologists and clinicians with an anatomical and tomographic map, integrating available literature, rather than to define the best positioning for the estimation of the interscapular region of the cat, in order to guide them toward a correct interpretation before submitting the animal to surgery, especially in case of FISS.

Finally, this issue was addressed ethically since, unlikely most of previous publications with comparable technical approaches, no animals were sacrificed to perform the investigation.

## Abbreviations

CT: Computed tomography
MRI: Magnetic resonance imaging
FISS: Feline injection-site sarcomas
M: Muscle
Mm: Millimeters
